# How does age affect changes in leg muscle activation patterns and leg joint moments during prolonged walking?

**DOI:** 10.1007/s00421-025-05867-2

**Published:** 2025-06-25

**Authors:** Yujin Kwon, Hoon Kim, Jason R. Franz

**Affiliations:** 1 Department of Rehabilitation Medicine, Seoul National University Hospital, Seoul, South Korea; 2 Department of Sports Medicine, Soonchunhyang University, Asan, South Korea; 3 Department of Software Convergence, Graduate School, Soonchunhyang University, Asan, South Korea; 4 Lampe Joint Department of Biomedical Engineering, University of North Carolina at Chapel Hill and North Carolina State University, Chapel Hill, NC USA

**Keywords:** Gait, Electromyography, Frequency, Fatigue, Fatigability, Biomechanics

## Abstract

The ways in which age affects neuromuscular control in response to walking-related fatigue are poorly understood. Better understanding of the consequences of walking-related fatigue can inform the development of strategies to improve independent mobility for older adults. In this study, we measured leg muscle excitations and net joint moments in younger and older adults during a 30-min walking trial. Twelve leg muscles were monitored, and wavelet transformation and principal component analyses quantified the effects of age and time on muscle excitation patterns. Perceived exertion increased in both age groups, with higher terminal values in older adults. Over the course of prolonged walking, mean EMG frequency and amplitude decreased while EMG intensities in the slower frequency ranges increased for soleus, lateral gastrocnemius, tibialis anterior, peroneus longus, and gluteus maximus muscles. For soleus muscle, a time-dependent decrease in mean frequency was observed only for older adults. We observed a distal-to-proximal redistribution of net joint moments during prolonged walking independent of age; however, older adults walked with greater peak hip joint moments than younger adults. Our results suggest that shank muscles may exhibit higher fatigability during prolonged walking, precipitating an increased demand on proximal leg muscles to power walking. Walking in older adults is often characterized by an increased reliance on proximal leg muscles, which has in turn been implicated in their higher metabolic cost of transport. Accordingly, our collective findings point to neuromuscular changes during prolonged walking that may cause older adults to be more susceptible to walking-related fatigue than younger adults.

**Supplementary Information:**

The online version contains supplementary material available at 10.1007/s00421-025-05867-2.

## Introduction

Identified as a risk factor during functional activities, muscle fatigue is important to quantify as it may prevent the generation of the requisite forces required for those tasks (Manty et al. 2012; Morrison et al. 2016). During functional activity, it is difficult to objectively measure fatigue via declines in maximum muscle force capacity. Thus, surrogates of muscle fatigue are common. Based on principles from muscle neurophysiology, surface electromyography (EMG) is a frequently used surrogate, with increased amplitude and decreased frequency of EMG signals indicating the progression of local muscle fatigue (Linssen et al. 1993; Kuorinka 1988; Esposito et al. 1998). The validity of these neuromuscular outcomes has been demonstrated for isolated single-joint movements (Bonato et al. 2003; González-Izal et al. 2010; Matsunaga et al. 2021). We recently extended the application of these surface EMG techniques to characterize neuromuscular markers of walking-related fatigue over the course of an ecologically-relevant 30-min walking period (Kwon et al. 2023). There, we used time–frequency analysis with wavelet transformation and principal component analysis to distinguish complex characteristics of neuromuscular changes and changes thereof following prolonged walking in younger adults. Prolonged walking is a uniquely prevalent daily activity participated in by people of all ages. However, although walking-related fatigue could be a critical determinant of community independence that may become worse in old age. As an important step toward understand those associations, it is necessary to investigate the time course of age-related differences neuromuscular control during prolonged walking.

Anecdotal evidence would imply that older adults are more susceptible to walking-related fatigue than younger adults. Age-related changes that could affect muscle fatigability include, for example, reduced muscle size and strength (Holloszy et al. 1995; Kent-Braun et al. 2000; Evans and Lexell 1995), diminished phosphorylation coupling (Fitzgerald et al. 2016), antagonist muscle coactivation (Franz and Kram 2013), and/or alterations in the muscles relied upon to power walking (Boyer et al. 2017). For a daily activity that is performed for prolonged periods of time (i.e., prolonged walking), older adults may show altered muscle recruitment patterns due to their decreased muscle power and increased fatigability with aging (McNeil and Rice 2007; Yoon et al. 2008). Many previous studies have evaluated the effect of muscle fatigue on gait biomechanics and reported that muscle fatigue is associated with reduced postural control, therefore implicating elevated fall risks for older adults (Parijat and Lockhart 2008; Granacher et al. 2010; Barbieri et al. 2014; Helbostad et al. 2007). However, these studies used tasks less relevant to daily life than walking to elicit fatigue, such as repetitive knee extension or sit-to-stand movements. Given its daily prevalence, prolonged walking may itself induce functionally-relevant levels of muscle fatigue—something we have shown previously in younger adults (Kwon et al. 2023). Given that older adults invest relatively higher levels of lower limb muscular effort in walking than younger adults (Hortobágyi et al. 2003), they may exhibit different patterns or faster rates of neuromuscular changes due to walking-related fatigue.

One specific example that could affect intermuscular differences in the time course of neuromuscular changes during prolonged walking with age is that, when compared with younger adults, older adults walk with diminished ankle plantarflexor power output (Kerrigan et al. 1998; Judge et al. 1996). To compensate for insufficient ankle push-off, older adults rely more on proximal muscles to power walking, exhibiting a well-known phenomenon deemed the ‘distal-to-proximal redistribution’ (DeVita and Hortobagyi 2000; Franz 2016). Unfortunately, due to their different muscle–tendon architecture, this distal-to-proximal redistribution has been implicated as a determinant of higher metabolic costs in older adults, which conceptually has the potential to accelerate fatigue (Fickey et al. 2018; Pimentel et al. 2021). Our previous work in younger adults found evidence that the distal leg muscles exhibit more pronounced neuromuscular changes associated with fatigue during prolonged walking (Kwon et al. 2023). Although net joint moments were not included in that work, we would thereby suspect: (1) an emergent reliance on proximal (e.g., hip joint) muscles with the accumulation of walking-related fatigue, which may (2) compound the habitual distal-to-proximal redistribution of older adults with important implications for neuromuscular changes during prolonged walking.

Understanding age-related differences in the neuromuscular changes associated with walking-related fatigue is an important step to broader efforts to maintain the independent mobility of older adults. Thus, the objective of this study was to quantify age-related differences in the time course of neuromuscular control during prolonged walking, which we would interpret in the context of walking-related fatigue. We first hypothesized that EMG activations in slower frequency ranges would increase over time, and that the magnitude of those increases would be more pronounced: (i) for distal than for proximal leg muscles and (ii) for older adults than for younger adults. We also hypothesized that these differences would be accompanied by a time-dependent redistribution in net joint moments, evidenced by decrease ankle joint moments and increased hip joint moments over time, with larger magnitudes in older adults.

## Methods

### Participants

Fifteen healthy younger adults (18–35 years) and fourteen older adults (> 65 years) participated (Table 1). Participants had no neurological impairment or leg injuries. Each participant provided written informed consent approved by the University of North Carolina at Chapel Hill Biomedical Sciences Institutional Review Board before participating. Before performing walking trials, each participant answered a questionnaire asking how many times on average they perform strenuous (heart beats rapidly; e.g., running, vigorous bicycling), moderate (not exhausting; e.g., fast walking, easy bicycling), or mild exercise (minimal effort; e.g., yoga, easy walking) for more than 15 min to contextualize their physical activity level. Although older adults tended to participate in lesser physical activity than younger adults, on average, these differences were not statistically significant.

**Table 1:** Participant demographics

	Younger adults (*n* = 15)	Older adults (*n* = 14)	*p* value
Age (years)	24.5 ± 4.6	73.3 ± 4.9	
Sex (female/male)	9/6	9/5	
Height (m)	1.74 ± 0.11	1.68 ± 0.09	0.172
Mass (kg)	71.9 ± 13.5	68.7 ± 15.4	0.562
Walking speed (m/s)	1.34 ± 0.11	1.20 ± 0.20	0.105
*Physical activity level (times per week)*			
Strenuous exercise	3.0 ± 1.7	2.1 ± 2.3	0.278
Moderate exercise	3.8 ± 2.4	2.6 ± 2.2	0.175
Mild exercise	3.8 ± 2.8	3.4 ± 2.7	0.669

Values reported in means ± SD

### Experimental procedure

We measured each participant’s self-selected walking speed as the average of three 30-m walks using two timing gates (Bower Timing Systems, Draper, UT, USA). Participants then walked on a dual-belt treadmill (Bertec Corp., Columbus, OH) at their preferred walking speed for a 5-min acclimation period. Then, they walked at 1.2 m/s for two minutes, which we later used as a baseline for EMG normalization (Kwon et al. 2023). Finally, in a prolonged walking trial, participants walked at their self-selected walking speed for 30 min.

### Measurements

Wireless surface electromyography (EMG) recorded muscle excitations of 12 dominant-leg muscles (Trigno, Delsys Inc., Natick, MA) at 1000 Hz. Leg dominance of each participant was defined by asking the leg they would use to kick a ball. The twelve muscles included: Shank (SOL, soleus; LGAS, lateral gastrocnemius; MGAS, medial gastrocnemius; TA, tibialis anterior; PL, peroneus longus), Thigh (VL, vastus lateralis; VM, vastus medialis; RF, rectus femoris; BIFEM, biceps femoris; ST, semitendinosus), and Hip (GMAX, gluteus maximus; GMED, gluteus medius). Before attaching electrodes to each muscle, the site was shaved if needed and wiped with alcohol to minimize impedance. All electrodes were placed on the respective muscle bellies according to the SENIAM guidelines (seniam.org). Synchronously, we collected the trajectories of 36 retroreflective markers placed on the trunk and lower extremities at 100 Hz using a 15-camera 3D motion capture system (Motion Analysis Corporation, Santa Rose, CA) and bilateral ground reaction force (GRF) data at 1000 Hz using instrumented dual-belt treadmill (Bertec Corp., Columbus, Ohio, USA). Anatomical markers were placed *bilaterally* on the acromion, posterior superior iliac spine, anterior superior iliac spine, lateral knee joint, lateral malleoli, heel, and 1st and 5th metatarsophalangeal joint, as well as on the 7th cervical and 10th thoracic vertebrae, the posterior sacrum, right scapula, suprasternal notch, and sternum. In addition, rigid plates with 3–4 tracking markers were placed on the lateral thighs and shanks. During the 30-min walking trial, participants verbally reported their rate of perceived exertion (RPE) on a 10-point Borg scale every ten minutes. We secured EMG sensors and markers with Coban self-adherent wrap (3 M, St. Paul, MN, USA) to prevent them from falling off during the prolonged walking trial.

### EMG analysis

Raw EMG signals were bandpass filtered between 20 and 400 Hz using a Butterworth 4th order filter. We then analyzed EMG signals in both time and frequency domains using wavelet transformations. The detailed method of the analysis is described in our previous papers (Kwon et al. 2023; Kim and Franz 2021). Briefly, we created 11 nonlinearly scaled Morlet wavelets (Cohen 2019) and converted each Morlet wavelet and EMG signals to the frequency domain with fast Fourier transforms (FFT). After multiplying them (i.e., Morlet wavelet and EMG signals) element by element, inverse FFT was used to calculate the EMG intensity data for each Morlet wavelet in the time domain. In the stacked EMG intensities of all wavelets, instantaneous mean frequency (${f}_{m}$) of each gait cycle was calculated (Eq. 1).

1 $${f}_{m,i}= \frac{{\sum }_{k=1}^{k=11}{cf}_{k}{I}_{k,i}}{{\sum }_{k=1}^{k=11}{I}_{k,i}}$$

where $m$ is the muscle, $k$ is the Morlet wavelet number (e.g., 1 ~ 11), $i$ is the time point of the gait cycle (e.g., 1 ~ 100), $cf$ is the central frequency of each wavelet, and $I$ is the EMG intensity. Finally, we calculated mean ${f}_{m}$ of the gait cycle by averaging all ${f}_{m,i}$.

Here, we distinguish “intensity” from “amplitude” to describe signal analyses in the frequency domain. For the EMG amplitude analysis, EMG signals underwent bandpass filtering, fully rectification and filtering with a 4th order Butterworth filter with a cutoff frequency of 10 Hz. The EMG intensities and amplitudes were normalized to 100% of the gait cycle. The normalized EMG intensity and amplitude data were then normalized to their mean values obtained from the first 1 min of the baseline walking trial. Finally, instantaneous mean frequency (${f}_{m}$) and the normalized amplitude data were averaged for four 1-min time blocks every ten minutes (i.e., 0–1 min; 9–10 min; 19–20 min; 29–30 min). All data analysis were conducted using a custom-written code in Matlab (MathWorks Inc., Natick, MA, USA).

### Net joint moments

The GRF and the marker data were filtered using a 4th order Butterworth filter with a cutoff frequency of 20 Hz and 6 Hz, respectively. The motion and GRF data were then imported and processed in OpenSim musculoskeletal modelling software (version 4.5) (Delp et al. 2007) to estimate lower limb joint net joint moments. To create each participants’ model, we first linearly scaled the segment lengths of the Gait2392 model using anthropometrics (height and body mass) and marker pairs for each body segment (e.g., lateral epicondyle and lateral malleolus for the tibia segment) obtained during a static trial. Joint angles were computed via an inverse kinematics approach that minimized the position error between the model’s and measured marker. Then, we used the inverse dynamics tool to calculate the net joint moments of the ankle and hip joints on the dominant leg, based on joint angles obtained from inverse kinematics and ground reaction force (GRF) data measured by the instrumented treadmill. To analyze emergence of a distal-to-proximal redistribution over time, we calculated peak ankle plantarflexor moments (distal joint) and peak hip flexor and extensor moments (proximal joint) for each stride. Finally, all moment data were averaged for all gait cycles taken within four 1-min time blocks every 10 min as we did for the EMG data.

### Statistical analysis

Principal component analysis (PCA) was used to reduce the dimensionality and capture patterns of variance of the high-dimensional EMG intensity data matrices (mean intensities of each muscle * 101 time points * 11 Morlet wavelets for 4 time blocks). By transforming the original complex dataset into a smaller set of principal components (PCs) that explain the most relevant patterns of variance, we can more effectively identify dominant activation trends and compare differences across conditions/populations. PCA calculated PCs and scores for PCs that had the biggest variance. The first three PCs were chosen which accounted for more than 5% of the total variance (VAF).

A two-way mixed ANOVA was performed on the following variables, with time as a within-subject repeated measure and age group as a between-subject factor. The effect of time and age group was tested on the variables: PC scores of each PC, mean EMG frequency, mean EMG amplitudes, net joint moments and RPE scores. To test the assumption of sphericity of the data, Maulchly’s test of sphericity was used. When the assumption was violated, a Greenhouse–Geisser correction was performed.

For significant main effects, the Bonferroni correction was used for pairwise comparisons. Participant demographics were compared using two sample *t* test. Therefore, the significance level was 0.05 for main effects and $\alpha$ = 0.0125 (0.05/4) for pairwise comparisons to control the experiment-wise error rate. We used R (Rstudio, Boston, MA) for all statistical analyses.

## Results

### Rate of perceived exertion

**Fig.1:**
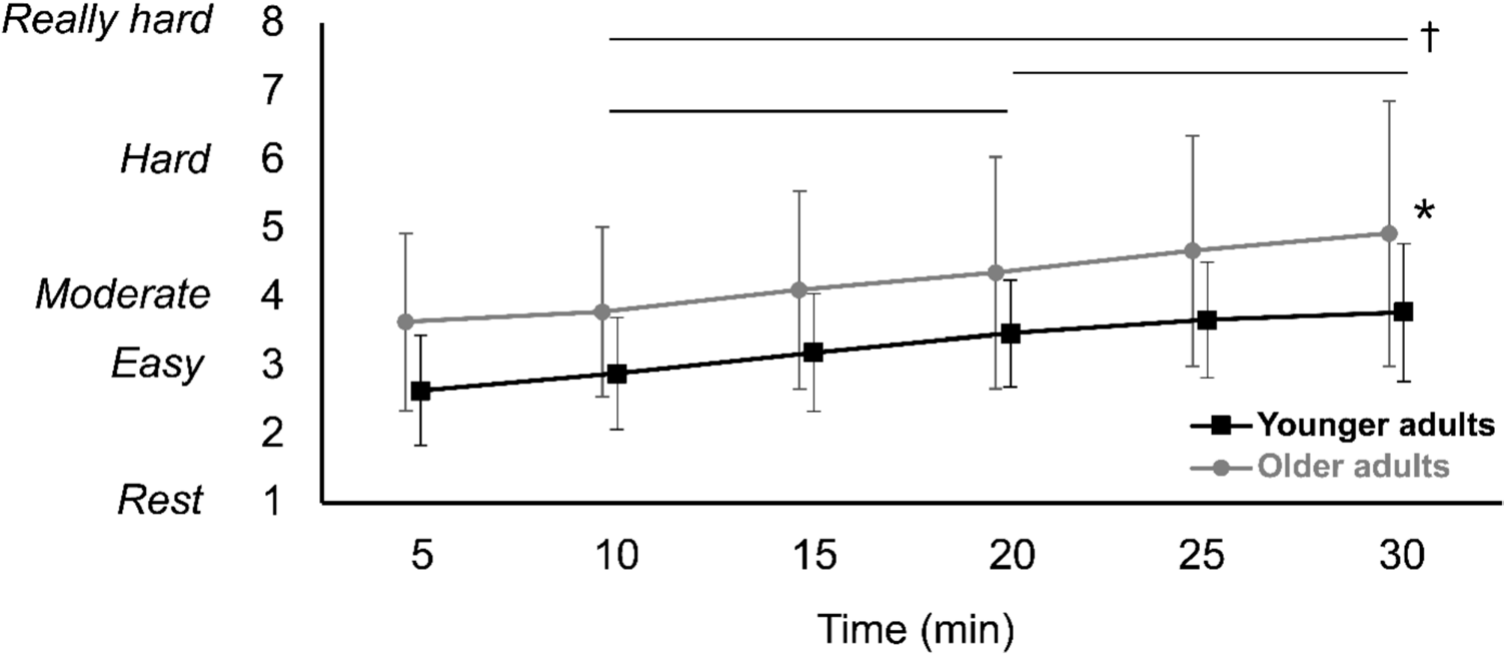
Rate of perceived exertion of younger and older adults. Although the scores were collected every 5 min, data at minute 10, 20, and 30 were used for consistency with other data analysis. Statistical markers (* and †) indicate significant group and time effects, respectively (*p* < 0.05),

RPE scores showed a significant time (*p* = 0.027) and group effect (*p* = 0.047), but no significant interaction (*p* = 0.275). Both groups showed a significant increase in RPE scores over time (younger adults: *p* = 0.001, older adults: *p* < 0.001), and older adults reported significantly higher RPE scores at minute 30 (*p* = 0.039) (Fig. 1).

### EMG frequency

**Fig. 2:**
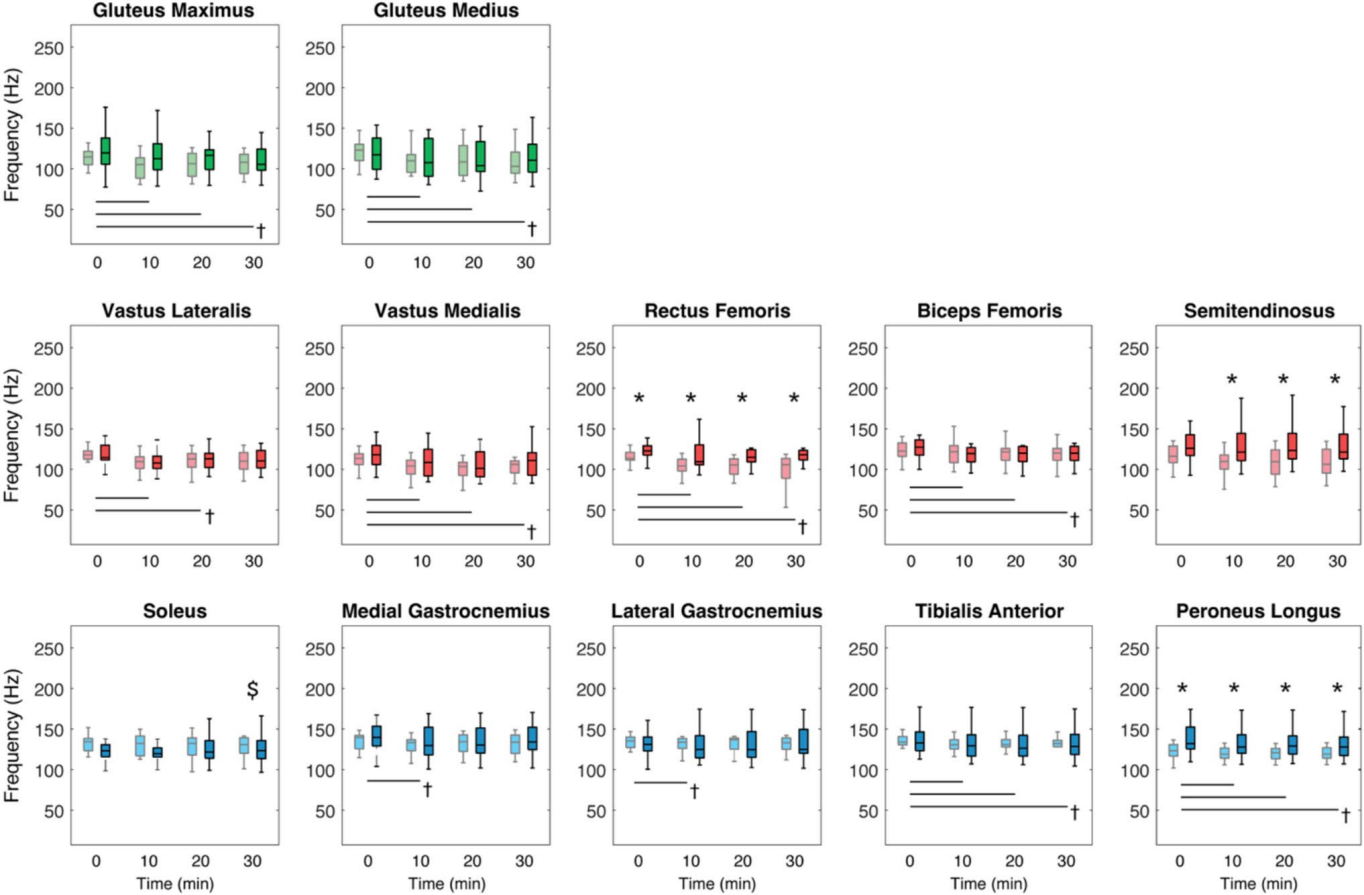
Instantaneous mean frequency of younger and older adults at 1-min time blocks every 10 min. Muscles are colored according to muscle group (shank: blue; thigh: red; hip: green) for older (light color; gray) and younger adults (dark color; black). Statistical markers (*, †, and $) indicate significant group, time, and interaction effects, respectively (*p* < 0.05), and horizontal bars indicate pairwise significant differences (*p* < 0.0125)

Figure 2 shows instantaneous mean frequency (${f}_{m}$) versus time during the 30-min walk. ${f}_{m}$ significantly decreased with time for all muscles (*p* < 0.05) except SOL and ST. A significant group effect was detected for PL (*p* = 0.019), RF (*p* = 0.008), and ST (*p* = 0.020) where older adults showed slower ${f}_{m}$ than younger adults. A significant interaction effect was found for SOL (*p* = 0.003) for which ${f}_{m}$ decreased significantly with time only for older adults after the Bonferroni correction (*p* = 0.012).

### EMG intensity

For the first three PCs that had VAF > 5%, we found significant group effects in PC scores for SOL, LGAS, TA, PL, BIFEM, and ST, significant time effects for all muscles except VL and ST, and significant group × time interaction effects for SOL, LGAS, TA, and GMED. After the Bonferroni correction, the group effect was significant for SOL, TA, PL, and BIFEM while the time effect was significant for SOL, LGAS, PL, BIFEM, and GMAX.

**Fig. 3:**
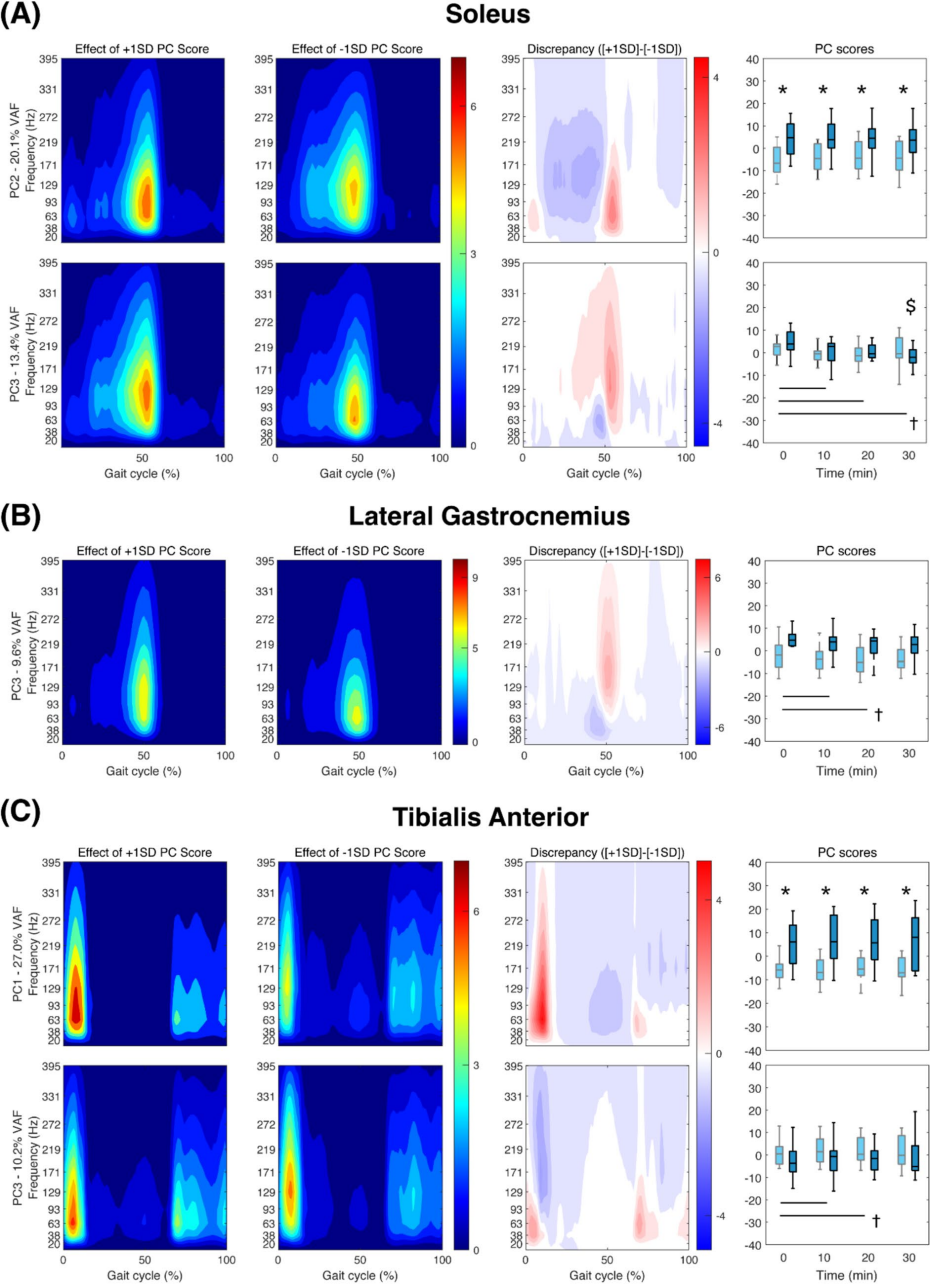
Muscle intensities of Soleus (**A**), Lateral Gastrocnemius (**B**), and Tibialis Anterior (**C**) of younger and older adults for which significant effects of age and time in PC scores were observed. The only PCs of each muscle that showed a significant difference after the Bonferroni correction were included in the figure. Heatmaps in the first two columns show the effect of + 1 and − 1 standard deviation (SD) PC score. Discrepancy plot shows the effect of SD on the muscle activations where high intensity in red areas mean higher PC scores and blue areas mean lower PC scores. In the box plots, statistical markers (*, †, and $) indicate significant group, time and interaction effects, respectively (*p* < 0.05) for older (light color; gray) and younger adults (dark color; black), and horizontal bars indicate pairwise significant differences (*p* < 0.0125)

**Fig. 4:**
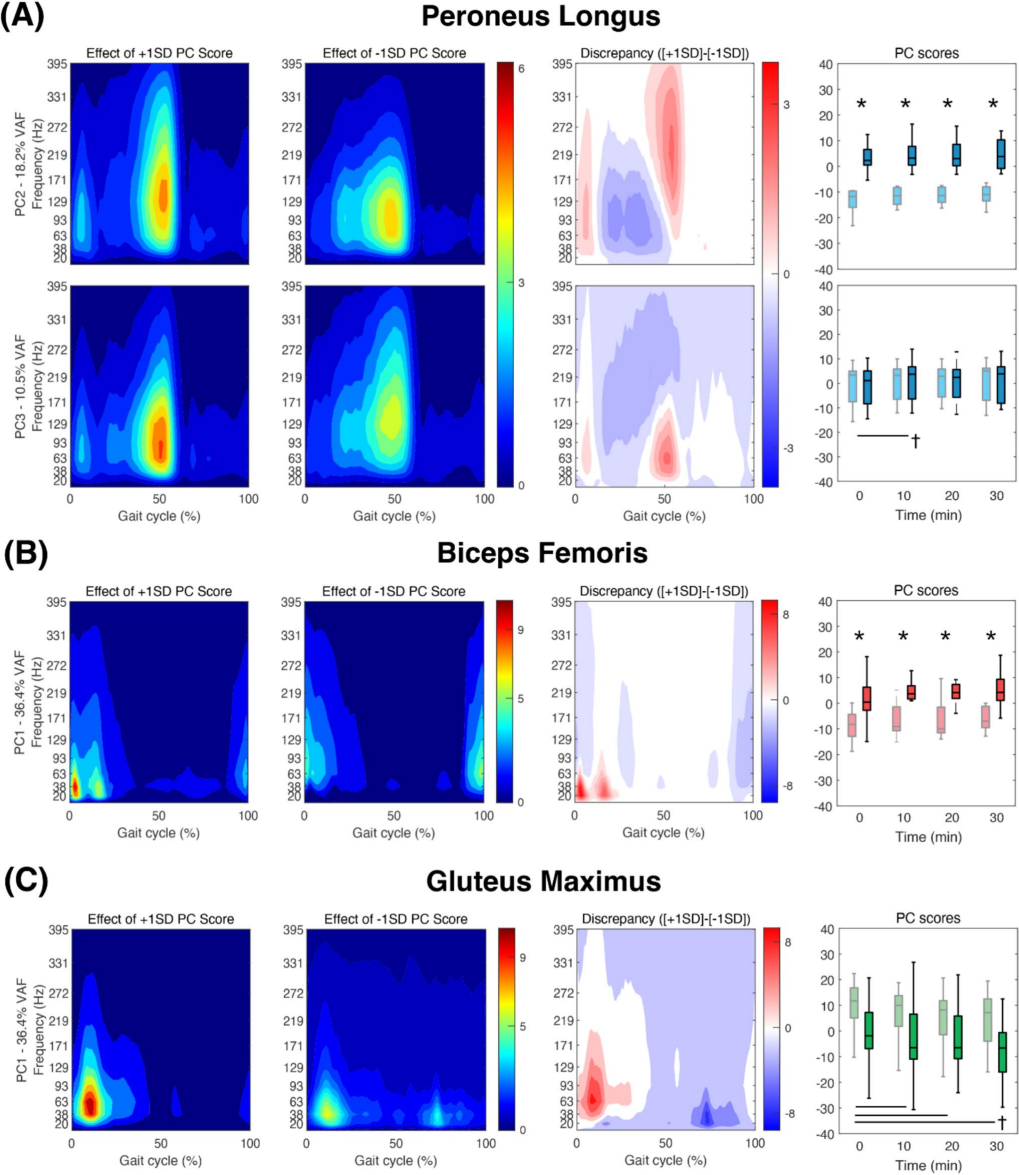
Muscle intensities of Peroneus Longus (**A**), Biceps Femoris (**B**), and Gluteus Maximus (**C**) of younger and older adults for which significant effects of age and time in PC scores were observed. The only PCs of each muscle that showed a significant difference after the Bonferroni correction were included in the figure. Heatmaps in the first two columns show the effect of + 1 and − 1 standard deviation (SD) PC score. Discrepancy plot shows the effect of SD on the muscle activations where high intensity in red areas mean higher PC scores and blue areas mean lower PC scores. In the box plots, statistical markers (*, †, and $) indicate significant group, time and interaction effects, respectively (*p* < 0.05) for older (light color; gray) and younger adults (dark color; black), and horizontal bars indicate pairwise significant differences (*p* < 0.0125)

The muscle intensity results are described in Figs. 3 and 4. In the first two columns, representative heatmaps of + 1 and − 1 standard deviation (SD) PC score are provided, showing the effect of PC score on muscle intensities in a gait cycle. Then, a discrepancy plot was generated by subtracting the second figure (− 1 SD) from the first figure (+ 1 SD) where the areas affected by higher PCs are indicated in red and those affected by lower PC scores are indicated in blue. Lastly, PC scores of younger and older adults at 1-min time blocks every 10 min are compared in the box plot.

For SOL, PC2 had a significant group effect at all time points, which was driven by greater intensities in all frequency ranges during mid-stance (20 ~ 40% gait cycle) for older than younger adults (Fig. 3A). Here, PC3 had a significant interaction effect where time effect was significant only for younger adults (*p* = 0.005). The time-dependent decrease in PC3 scores driven by greater intensities in slower frequency ranges during push-off (around 50% of gait cycle). For LGAS, PC3 captured an increase in the intensities in slow-to-mid frequency ranges throughout the gait cycle for minutes 10 and 20 (Fig. 3B). TA had a significant group effect on PC1, where older adults walked with greater intensities than younger adults in all frequency ranges during late stance (40 ~ 60% gait cycle) (*p* < 0.01). Independent of age, PC3 captured increased TA intensities during early stance (0 ~ 10% gait cycle) at minutes 10 and 20 (*p* < 0.01) (Fig. 3C). For PL, PC2 scores showed a significant group effect at all time points (*p* < 0.001), which was driven by greater intensities in slow-to-mid frequency ranges during mid-stance for older adults compared to younger adults. PC3 captured increased PL intensities in slow-to-mid frequency ranges during push-off at minute 10, independent of age (*p* < 0.001) (Fig. 4A). For BIFEM, a significant group effect in PC1 scores at all time points (*p* < 0.05) was driven by greater intensities in the slower frequency ranges prior to heel contact (90 ~ 100% gait cycle) for older adults as compared to younger adults (Fig. 4B). GMAX showed a significant time-dependent decrease in PC1 scores from minute 0 to minute 10, 20, and 30 (*p* < 0.01) where intensities in all frequency ranges increased throughout the swing phase (Fig. 4C).

### EMG amplitude

**Fig. 5:**
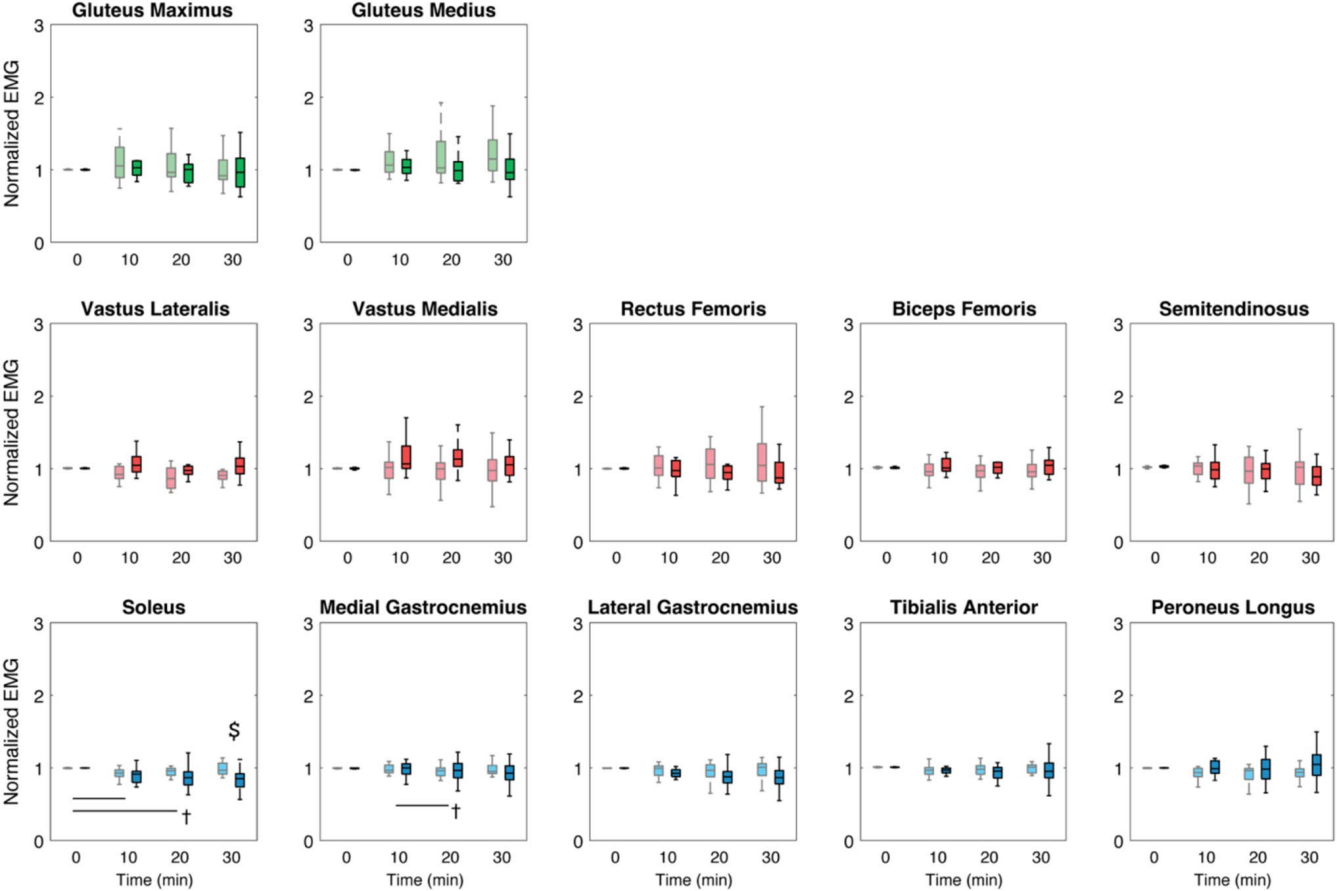
Mean EMG amplitudes of younger and older adults at 1-min time blocks every 10 min. Muscles are colored according to each muscle group (shank: blue; thigh: red; hip: green) for older (light color; gray) and younger adults (dark color; black). Statistical markers (*, †, and $) indicate significant group, time and interaction effects, respectively (*p* < 0.05), and horizontal bars indicate pairwise significant differences (*p* < 0.0125)

We found a significant main effect of time for the mean amplitude of SOL (*p* = 0.001) and MGAS (*p* = 0.024), and a significant group × time interaction effect for SOL (*p* = 0.019). After Bonferroni’s correction, a significant decrease over time in SOL mean amplitude was detected only for younger adults (*p* = 0.003) (Fig. 5).

### Net ankle and hip joint moments

**Fig. 6:**
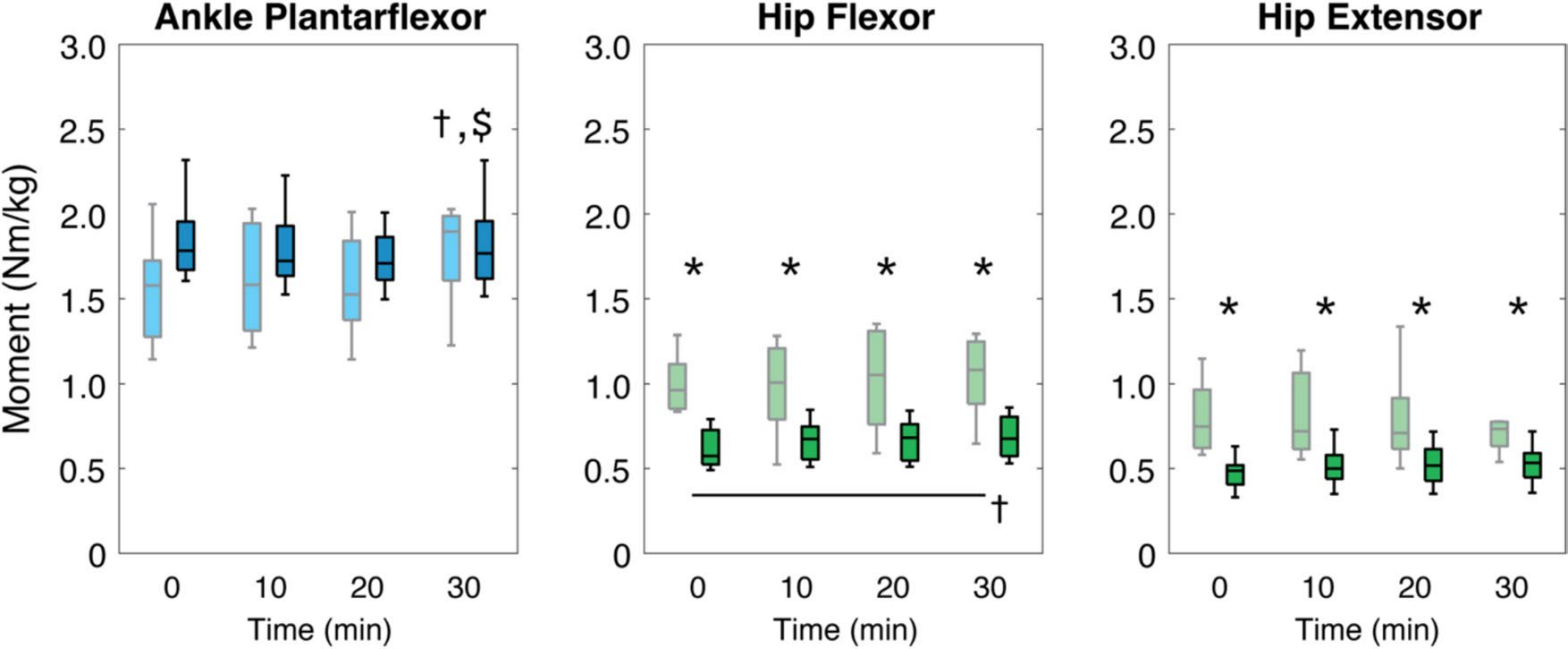
Mean net ankle and hip joint moments of younger and older adults at 1-min time blocks every 10 min. Muscles are colored according to each muscle group (shank: blue; thigh: red; hip: green) for older (light color; gray) and younger adults (dark color; black). Statistical markers (*, †, and $) indicate significant group, time and interaction effects, respectively (*p* < 0.05), and horizontal bars indicate pairwise significant differences (*p* < 0.0125)

Throughout the 30-min walking trial, hip flexor moments increased significantly with time (*p* < 0.001), and older adults walked with greater hip flexor and extensor moments compared to younger adults (*p* < 0.001) (Fig. 6). For ankle plantarflexor moment, a time main effect (*p* = 0.003) was detected although it was no longer significant after the Bonferroni correction. A significant group × time interaction (*p* = 0.002) for ankle plantarflexor moment was detected, however the time effect was no longer significant for both age groups after the Bonferroni correction.

## Discussion

This study aimed to investigate the effect of age on neuromuscular characteristics and net joint moments during a 30-min bout of walking in the context of walking-related fatigue. During prolonged walking, perceived exertion significantly increased, reaching challenging levels at the end of the walking bout for older adults. Consistent with our previous finding, we found a generalized decrease in EMG mean frequency and amplitude during prolonged walking. We accept our first hypothesis that EMG intensities in the slower frequency ranges would increase with time was accepted for SOL, LGAS, TA, PL, and GMAX. We also accept our second hypothesis that hip joint moments would increase over time and would be greater for older than for younger adults. Together, we interpret our results to suggest that shank muscles may exhibit higher fatigability during prolonged walking, precipitating an increased demand on proximal leg muscles to power walking. As we describe in more detail below, our collective findings point to specific neuromuscular changes during prolonged walking durations that may cause older adults to be more susceptible to walking-related fatigue than younger adults.

We conducted time–frequency analyses with wavelet transformation to detect changes in muscle activation patterns with time that can be overlooked when analyzed with an aggregate measure such as mean frequency. We extend our previous findings to show that both younger and adult adults exhibit significant time-dependent changes in EMG intensities for SOL, LGAS, TA, PL, and GMAX (Kwon et al. 2023). For example, EMG intensities of SOL, LGAS, and PL increased in the slow-to-mid frequency ranges during push-off, while EMG intensities for TA increased in the slow-to-mid frequency ranges during early stance (Fig. 3). EMG intensities for GMAX also increased in the slow frequency ranges in the swing phase (Fig. 4). Our time–frequency analysis captured increases in muscle activations over time that were missed using mean EMG amplitude alone. We also captured significant age effects in EMG intensities for SOL, TA, PL, and BIFEM. Specifically, our results showed that older adults walked with a different recruitment strategy across the entire prolonged walking trial. Older adults walked with increased mid-stance EMG intensities for SOL and PL in all frequency ranges before they are called upon to enhance ankle plantarflexion during push-off. There, increased TA EMG intensities in all frequency ranges during push-off likely imply that older adults increase antagonist muscle coactivation—potentially to maintain ankle joint stability and/or mechanical impedance (Baratta et al. 1988; Solomonow et al. 1988; Iwamoto et al. 2017). Taken together, the group and time effects evident in our results suggest that the time–frequency analysis enables comprehensive interpretation of both time-dependent changes and age-related differences in neuromuscular characteristics of walking-related fatigue.

As a complement to our time–frequency analyses, we also quantified EMG mean frequency and amplitude to assess age-related differences in the development of muscle fatigue during prolonged walking. In our previous study in younger adults (Kwon et al. 2023), we suggested that walking-related fatigue may develop faster for distal leg muscles, which might be due to intermuscular differences in fiber composition (Johnson et al. 1973; Edström and Kugelberg 1968). Here, we found that EMG mean frequency: (i) decreased with time for all muscles except SOL and ST and (ii) decreased with time for SOL only for older adults (Fig. 2). Although it has been suggested that fatigue may develop faster for older adults than younger adults (Melzer et al. 2000; Boccia et al. 2016; Oliveira et al. 2017), the results of this study suggest that older adults might be more susceptible, especially to distal leg muscle fatigue during prolonged walking than younger adults. However, in contrast to the previous reports that concluded the development of muscle fatigue from EMG via decreased frequency and increased amplitude from single-joint movements (Luttmann et al. 2000), our decreases in mean frequency were accompanied by decreased amplitudes. We suspect that this difference arises from the ability of our participants to vary muscle force output as needed, whereas previous studies prescribed fixed force levels during isolated single-joint movements (Linssen et al. 1993; Kuorinka 1988; Esposito et al. 1998). Conversely, our participants walked at the same fixed speed but the relative contribution from individual muscles could vary with the potential accumulation of walking-related fatigue. This is supported by our results that EMG amplitudes of thigh and hip muscles tended to increase over time while the amplitudes of shank muscles decreased (Fig. 5). This may imply a distal-to-proximal shift of muscle recruitment, but another surrogate for muscle demand needs to be considered to better understand the phenomenon, motivating our inclusion of net joint moments. 

Our interpretation that distal leg muscles disproportionately succumb to walking-related fatigue in older and younger adults would imply a reduction in their force-generating capacity that would require compensation from more proximal leg muscles. This overall interpretation is supported by our results that, independent of age, peak hip flexor moments increased over time during prolonged walking (Fig. 6). In addition, and consistent with prior biomechanical studies (Savelberg et al. 2007; Kulmala et al. 2014), our older adults walked with a greater hip flexor and extensor moments than younger adults at all time points. Although not significant, smaller ankle moments of older adults were accompanied by greater hip moments, which reflects higher relative reliance on proximal leg muscles compared to younger adults. Together with the time-dependent increase in hip flexor moments of older adults, we interpret our results to suggest that waking-related fatigue precipitates a distal-to-proximal redistribution of leg muscle function, which disproportionately compounds an already heightened reliance on proximal leg muscles during walking for older adults (Cofré et al. 2011; DeVita and Hortobagyi 2000). The increased reliance on proximal leg muscles then can contribute to elevated metabolic cost, which could in turn accelerate the functional limitations caused by walking-related fatigue (Pimentel et al. 2021). This important take-home message may be used to guide muscle-specific training interventions and/or the development of walking assistive devices aimed at reducing walking-related muscle fatigue. Specifically, training interventions may focus on improving fatigue-resistance of the distal leg muscles, while assistive devices may focus on mitigating time-dependent increases in hip flexor moments during the push-off phase to promote improved walking capacity for older adults.

There are some limitations in this study. Since there has been a lack of studies that evaluated muscle fatigue in dynamic situations such as walking, we cannot conclude that our results indicate muscle fatigue developed from 30-min walking. Nevertheless, we interpret our results as neuromuscular adaptation during prolonged walking in the context of walking-related fatigue, while we do not know the relative effect of central versus peripheral alterations leading to the measured changes in EMG. Although we observed decreased mean frequency, we did not observe increased amplitude—hallmark neuromuscular markers of muscle fatigue when observed in concert. Although we provide a reasonable explanation above, an alternative interpretation is that our 30-min walking duration was insufficient to induce fatigue. However, our older adults reported higher RPE scores than younger adults, and the score reached 5 (challenging) at the end of the prolonged walking trial (Fig. 1). A longer duration might be selected for future studies, but a reasonable duration for older adults needs to be chosen considering their differences in physical capacity. While a few older adults who could not walk on a treadmill without holding the front bar were excluded, some of the included older adults were not able to walk on a treadmill as fast as they were in the hallway. Furthermore, different gait adaptations, such as changes in stride length or cadence might occur during overground prolonged walking (Barbieri et al. 2014), although we did not observe significant changes in stride length over time in our treadmill walking study (Table S5). Thus, the extent to which our results from treadmill walking generalize to overground remains unclear and may be the focus of future studies. We also acknowledge that any biomechanical model used to estimate joint kinematics and kinetics are subject to errors in, for example, anthropometric assumptions, error minimization/cost functions, and kinematic constraints.

## Conclusion

Young and older adults exhibited different neuromuscular changes during the course of prolonged walking which we interpreted in the context of walking-related fatigue. The time–frequency analysis using wavelet transformation captured age-related differences in EMG intensities across several frequency ranges which would have been otherwise missed using mean frequency and/or amplitude alone. The age-related differences in fatigability we report—particularly those revealing a distal-to-proximal redistribution with time—are likely to exacerbate hallmark age-related changes in habitual muscle recruitment during walking and may disproportionately affect independent mobility in older adults. Collectively, understanding differences in neuromuscular characteristics between age groups may help us develop fatigue monitoring and walking assistive devices to mitigate walking-related fatigue and maintain independent mobility for older adults. For example, real-time visual biofeedback may be used during prolonged walking to mitigate the distal-to-proximal redistribution, which has shown its effect during shorter walking bouts in older adults (Browne and Franz 2019). Future studies may also explore the effect of resistance training to improve shank muscle strength on delaying muscle fatigue and attenuating the distal-to-proximal redistribution.

## Supplementary Information

Below is the link to the electronic supplementary material.

Supplementary file1 (DOCX 38 KB)

## Data Availability

Data will be available on reasonable request.

## Abbreviations

ANOVA: Analysis of variance
BIFEM: Biceps femoris
CoM: Center of mass
EMG: Electromyography
FFT: Fast Fourier transforms
PL: Peroneus longus
GMAX: Gluteus maximus
GMED: Gluteus medius
GRF: Ground reaction force
LGAS: Lateral gastrocnemius
MGAS: Medial gastrocnemius
PC: Principal component
PCA: Principal component analysis
RF: Rectus femoris
RPE: Rate of perceived exertion
ST: Semitendinosus
SOL: Soleus
TA: Tibialis anterior
VAF: Variance accounted for
VL: Vastus lateralis
VM: Vastus medialis
