# Supplementary material for: How does age affect changes in leg muscle activation patterns and leg joint moments during prolonged walking?
Source: Eur J Appl Physiol. 2025 Jun 25;125(12):3651–62. doi: 10.1007/s00421-025-05867-2 (PMC12678509; doi:10.1007/s00421-025-05867-2)
Supplement: Supplementary file 1 — Supplementary file1 (DOCX 38 KB) [file 421_2025_5867_MOESM1_ESM.docx]

**Table S1** F-values and p-values of two-way mixed ANOVA for the slope of instantaneous mean frequency over prolonged walking trial. Asterisks (*) indicate a significant effect (p<0.05).

| Muscles | F/p-values | Group | Time | Group*Time |
| --- | --- | --- | --- | --- |
| SOL | F | 0.983 | 0.465 | 5.077 |
|  | P | 0.330 | 0.708 | 0.003* |
| MGAS | F | 0.131 | 6.834 | 0.214 |
|  | P | 0.720 | <0.001* | 0.887 |
| LGAS | F | 0.086 | 5.148 | 0.867 |
|  | P | 0.771 | 0.003* | 0.462 |
| TA | F | 0.093 | 21.723 | 0.334 |
|  | P | 0.763 | <0.001* | 0.801 |
| FL | F | 6.272 | 7.395 | 0.318 |
|  | P | 0.019* | <0.001* | 0.812 |
| VL | F | 0.714 | 3.867 | 0.707 |
|  | P | 0.406 | 0.012* | 0.551 |
| VM | F | 1.243 | 14.018 | 0.239 |
|  | P | 0.275 | <0.001* | 0.869 |
| RF | F | 8.195 | 12.170 | 1.646 |
|  | P | 0.008* | <0.001* | 0.185 |
| BIFEM | F | 0.522 | 11.657 | 0.225 |
|  | P | 0.476 | <0.001* | 0.878 |
| SETEN | F | 6.145 | 1.877 | 1.912 |
|  | P | 0.020* | 0.140 | 0.134 |
| GMAX | F | 0.387 | 10.122 | 0.349 |
|  | P | 0.539 | <0.001* | 0.790 |
| GMED | F | 0.106 | 9.793 | 1.670 |
|  | P | 0.747 | <0.001* | 0.180 |

**Table S2** F-values and p-values of two-way mixed ANOVA for PC scores. Asterisks (*) indicate a significant difference.

| Muscles | PC | F/p-values | Group | Time | Group*Time |
| --- | --- | --- | --- | --- | --- |
| SOL | PC1 | F | 3.121 | 2.127 | 3.812 |
|  |  | P | 0.090 | 0.104 | 0.013 |
|  | PC2 | F | 9.391 | 1.222 | 2.033 |
|  |  | P | 0.005* | 0.308 | 0.116 |
|  | PC3 | F | 0.007 | 6.342 | 3.179 |
|  |  | P | 0.932 | <0.001* | 0.029* |
| MGAS | PC1 | F | 2.591 | 2.311 | 1.207 |
|  |  | P | 0.120 | 0.083 | 0.313 |
|  | PC2 | F | 0.009 | 1.303 | 1.468 |
|  |  | P | 0.926 | 0.279 | 0.230 |
|  | PC3 | F | 0.220 | 3.281 | 0.768 |
|  |  | P | 0.643 | 0.025* | 0.515 |
| LGAS | PC1 | F | 0.946 | 3.603 | 2.385 |
|  |  | P | 0.340 | 0.017* | 0.076 |
|  | PC2 | F | 5.451 | 2.325 | 2.949 |
|  |  | P | 0.028* | 0.081 | 0.038* |
|  | PC3 | F | 6.209 | 5.668 | 0.628 |
|  |  | P | 0.019* | 0.001* | 0.599 |
| TA | PC1 | F | 13.370 | 0.596 | 1.805 |
|  |  | P | 0.001* | 0.620 | 0.153 |
|  | PC2 | F | 2.005 | 0.570 | 2.937 |
|  |  | P | 0.169 | 0.636 | 0.038* |
|  | PC3 | F | 1.690 | 4.545 | 0.030 |
|  |  | P | 0.205 | 0.005* | 0.993 |
| FL | PC1 | F | 0.346 | 0.358 | 0.325 |
|  |  | P | 0.562 | 0.784 | 0.807 |
|  | PC2 | F | 29.992 | 5.098 | 0.296 |
|  |  | P | <0.001* | 0.003 | 0.828 |
|  | PC3 | F | 0.006 | 5.775 | 0.346 |
|  |  | P | 0.939 | 0.001* | 0.792 |
| VL | PC1 | F | 1.285 | 1.819 | 1.345 |
|  |  | P | 0.268 | 0.151 | 0.266 |
|  | PC2 | F | 0.201 | 2.692 | 1.876 |
|  |  | P | 0.658 | 0.052 | 0.141 |
|  | PC3 | F | <0.001 | 2.473 | 0.759 |
|  |  | P | 0.984 | 0.068 | 0.521 |
| VM | PC1 | F | 2.067 | 3.684 | 2.610 |
|  |  | P | 0.162 | 0.015* | 0.057 |
|  | PC2 | F | 0.075 | 4.428 | 0.373 |
|  |  | P | 0.786 | 0.006* | 0.773 |
|  | PC3 | F | 2.866 | 3.234 | 0.384 |
|  |  | P | 0.102 | 0.027* | 0.765 |
| RF | PC1 | F | 2.827 | 3.380 | 0.309 |
|  |  | P | 0.107 | 0.023* | 0.819 |
|  | PC2 | F | 0.928 | 0.270 | 0.805 |
|  |  | P | 0.346 | 0.847 | 0.496 |
|  | PC3 | F | 1.729 | 4.483 | 2.234 |
|  |  | P | 0.202 | 0.006* | 0.092 |
| BIFEM | PC1 | F | 6.801 | 3.863 | 1.211 |
|  |  | P | 0.015* | 0.013* | 0.312 |
|  | PC2 | F | 0.295 | 0.035 | 2.233 |
|  |  | P | 0.592 | 0.991 | 0.092 |
|  | PC3 | F | 0.019 | 0.289 | 0.374 |
|  |  | P | 0.892 | 0.833 | 0.772 |
| SETEN | PC1 | F | 3.031 | 1.663 | 2.610 |
|  |  | P | 0.094 | 0.183 | 0.058 |
|  | PC2 | F | 0.022 | 2.433 | 0.425 |
|  |  | P | 0.884 | 0.072 | 0.735 |
|  | PC3 | F | 4.290 | 0.483 | 0.188 |
|  |  | P | 0.049* | 0.695 | 0.905 |
| GMAX | PC1 | F | 4.008 | 8.457 | 1.288 |
|  |  | P | 0.057 | <0.001* | 0.285 |
|  | PC2 | F | 0.128 | 2.145 | 0.104 |
|  |  | P | 0.724 | 0.102 | 0.958 |
|  | PC3 | F | 1.437 | 2.409 | 0.418 |
|  |  | P | 0.242 | 0.074 | 0.741 |
| GMED | PC1 | F | 1.107 | 2.855 | 1.180 |
|  |  | P | 0.302 | 0.042* | 0.323 |
|  | PC2 | F | 0.783 | 2.782 | 0.305 |
|  |  | P | 0.384 | 0.046* | 0.822 |
|  | PC3 | F | 3.718 | 0.482 | 3.959 |
|  |  | P | 0.065 | 0.696 | 0.011* |

**Table S3** F-values and p-values of two-way mixed ANOVA for the mean EMG amplitudes. Asterisks (*) indicate a significant effect (p<0.05).

| Muscles | F/p-values | Group | Time | Group*Time |
| --- | --- | --- | --- | --- |
| SOL | F | 0.843 | 5.759 | 3.483 |
|  | P | 0.366 | 0.001* | 0.019* |
| MGAS | F | 0.321 | 3.291 | 0.712 |
|  | P | 0.575 | 0.024* | 0.547 |
| LGAS | F | 1.217 | 4.538 | 1.370 |
|  | P | 0.279 | 0.005 | 0.257 |
| TA | F | 0.27 | 2.349 | 1.165 |
|  | P | 0.590 | 0.078 | 0.327 |
| FL | F | 2.770 | 1.065 | 1.851 |
|  | P | 0.107 | 0.368 | 0.144 |
| VL | F | 1.429 | 1.231 | 0.914 |
|  | P | 0.241 | 0.303 | 0.438 |
| VM | F | 1.947 | 2.066 | 1.325 |
|  | P | 0.173 | 0.110 | 0.271 |
| RF | F | 0.438 | 0.525 | 0.501 |
|  | P | 0.514 | 0.666 | 0.683 |
| BIFEM | F | 1.860 | 0.684 | 1.329 |
|  | P | 0.183 | 0.564 | 0.270 |
| SETEN | F | 0.603 | 2.089 | 0.807 |
|  | P | 0.444 | 0.108 | 0.494 |
| GMAX | F | 0.601 | 2.045 | 0.327 |
|  | P | 0.445 | 0.113 | 0.806 |
| GMED | F | 2.521 | 2.177 | 2.512 |
|  | P | 0.123 | 0.096 | 0.064 |

**Table S4** F-values and p-values of two-way mixed ANOVA for joint moments. Asterisks (*) indicate a significant effect (p<0.05).

| Joint moments | F/p-values | Group | Time | Group*Time |
| --- | --- | --- | --- | --- |
| Ankle plantarflexor | F | 4.406 | 7.784 | 8.723 |
|  | P | 0.050 | <0.001* | <0.001* |
| Hip flexor | F | 35.655 | 5.552 | 0.730 |
|  | P | <0.001* | 0.002* | 0.539 |
| Hip extensor | F | 10.713 | 0.804 | 0.373 |
|  | P | 0.004* | 0.497 | 0.773 |

**Table S5** Stride length of younger and older adults at 1-min time blocks every 10 minutes (Means ± SD) and F-values and p-values of two-way mixed ANOVA. Asterisks (*) indicate a significant effect (p<0.05).

| Stride Length | 0 min | 10 min | 20 min | 30 min |
| --- | --- | --- | --- | --- |
| Younger adults | 1.39 ± 0.11 | 1.33 ± 0.06 | 1.35 ± 0.04 | 1.33 ± 0.04 |
| Older adults | 1.26 ± 0.16 | 1.30 ± 0.05 | 1.34 ± 0.03 | 1.35 ± 0.03 |
|  |  |  |  |  |
| *ANOVA Results* | F/p-values | Group | Time | Group*Time |
|  | F | 3.089 | 1.674 | 7.696 |
|  | P | 0.090 | 0.179 | <0.001* |

* The interaction effect was no longer significant after the Bonferroni correction.
